# Intravascular ultrasound to guide the management of intracoronary thrombus: a Case Report

**DOI:** 10.1186/1476-7120-2-18

**Published:** 2004-10-04

**Authors:** Italo Porto, Andrew RJ Mitchell, Vaishali Ashar, Adrian P Banning

**Affiliations:** 1 Cardiology Department, John Radcliffe Hospital, Oxford, UK

**Keywords:** intravascular ultrasound, thrombus, stent

## Abstract

Percutaneous coronary intervention can be associated with distal embolization of thrombotic material causing myocardial necrosis and infarction.

We discuss the role of intravascular imaging to guide the use of a distal protection device by describing the outcome of a young woman presenting with non-ST elevation myocardial infarction. Coronary angiography demonstrated an isolated minor stenosis in the proximal left anterior descending coronary artery with slight haziness beyond the lesion. Intravascular ultrasound confirmed an extensive thrombus overlying a bulky atherosclerotic plaque. A distal filter wire was therefore successfully used to reduce the risk of distal embolization.

The use of intravascular ultrasound in patients presenting with acute coronary syndrome may reveal large thrombi that are difficult to image using conventional angiographic techniques. Intravascular ultrasound can therefore be used as a tool to select lesions requiring distal protection.

## Background

Patients with acute coronary syndromes (ACS) are increasingly treated with an early invasive strategy. During percutaneous coronary interventions (PCI) interventionalists have often to deal with thrombus-laden lesions in native coronary vessels. This poses the serious problem of preventing macroscopic and microscopic distal embolization [1]. There have been many recent advances in this field, and new tools are available for shielding the distal microvasculature, including occlusive systems and basket filters. In the very emboli-prone saphenous vein grafts interventions, for example, both techniques have been associated with favourable results [2,3].

In native vessels cost and efficacy [4] considerations prompt a careful use of these devices, and a sensible approach could be selecting lesion and patients at high risk of distal embolization. For example, preventing distal embolization is particularly important in young patients presenting with their first coronary event with a large thrombotic burden [5,6].

Angiography alone is known to underestimate the risk of distal embolization: for example, only overt signs of massive thrombus burden are predictive of no-reflow in myocardial infarction patients treated with primary PCI [7]. Intravascular ultrasound (IVUS) has the potential to overcome many of the limitations of angiography, including lesion characterization and assessment of plaque rupture and thrombus [8-11], but its use is still restricted.

We present a case of successful prevention of macroscopic distal embolism in a young patient with ACS obtained combining IVUS and distal filter protection.

## Case presentation

A 41-year-old lady presented with a short history of cardiac sounding chest pain. She was a smoker, had hypertension and had a strong family history of premature ischaemic heart disease. The ECG showed widespread anterior T wave inversion. Conventional treatment was started immediately for ACS including intravenous nitrates, low molecular weight heparin, aspirin and clopidogrel. The patient remained pain-free following admission and serial biochemistry demonstrated a rise in cardiac troponin I to 24 mmol/l at 24 hours. There was no evidence of Q waves on the ECG and left ventricular function was normal on echocardiography.

**Figure 1:**
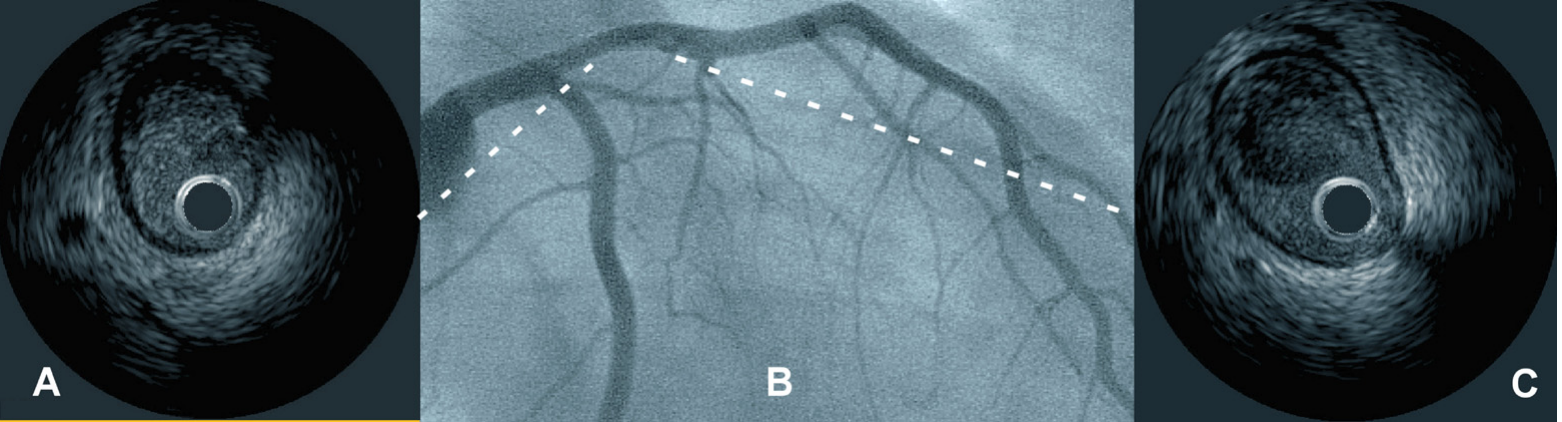
Composite image showing an angiographic right anterior oblique projection of the left coronary system (Panel B) and two intravascular ultrasound images (Panels A&C) before the intervention. Dotted line shows the approximate location of the IVUS slices. An eccentric plaque is visible in the proximal left anterior descending artery (Panel B), with some haziness at the distal end. IVUS confirms the presence of the plaque (Panel A) and shows a bulky, partially organized, thrombus (Panel C) loosely attached to the distal end of the plaque. No signs of plaque rupture are visible.

Coronary angiography was undertaken four days after admission. This demonstrated mural thickening of the proximal left anterior descending (LAD) coronary artery with a possible filling defect at the distal end of the lesion but no evidence of coronary atherosclerosis elsewhere (Fig. 1B). Interrogation of the proximal LAD stenosis with IVUS (Galaxy II system; Atlantis 40 MHz Catheter, Boston Scientific/Scimed, Inc., Maple Grove, Minnesota) showed a large intracoronary thrombus (Fig. 1C) adherent to a mural atheromatous plaque starting at the ostium of LAD (Fig. 1A). The thrombus had a lobulated appearance with no evidence of internal blood flow or speckling. It contained echolucent areas indicating cavitation and consistent with ongoing thrombus organization. No evidence of complete plaque rupture (intraplaque cavity communicating with the lumen) was seen.

**Figure 2:**
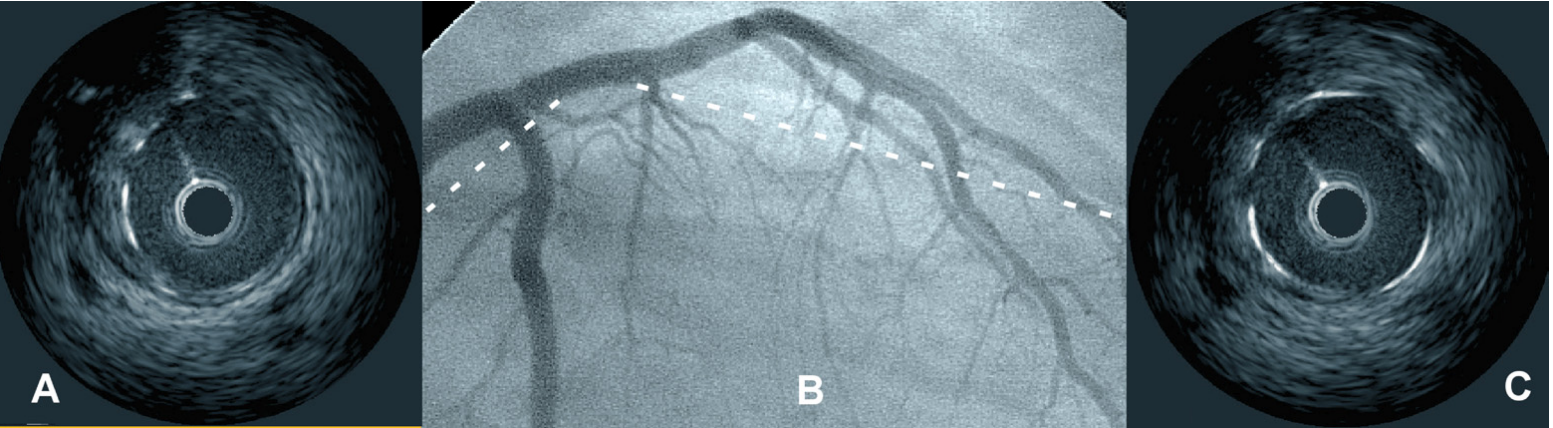
Composite image showing an angiographic right anterior oblique projection of the left coronary system (Panel B) and two intravascular ultrasound images (Panels A&C) after the intervention. Dotted line shows the approximate location of the IVUS slices. An excellent angiographic result is visible on the left anterior descending (Panel A). IVUS confirms good apposition of the stent (Panel A&B), with no residual or prolapsed thrombus.

Percutaneous coronary intervention was performed using distal filter protection with the EZ Filterwire (Boston Scientific, Natick, MA, USA). The GpIIb/IIIa antagonist Abciximab was administered. A 4.0 × 15 mm stent was placed directly with good final angiographic result (Fig. 2B) and no evidence of macroscopic distal embolisation. Repeat IVUS confirmed good apposition of the stent and the absence of residual thrombus (Fig. 2A&2C). At retrieval, the Filterwire contained a small but significant amount of pink thrombotic material and yellowish plaque debris. Equivalent chest x-ray radiation dose (assuming a single posteroanterior projection chest x-ray is eight centi-Gray/cm2) was 380.

No further Troponin I elevation occurred; the patient was discharged home the next day, and remains asymptomatic at two months.

## Discussion

Acute coronary syndromes with extensive thrombosis in the absence of widespread coronary atheroma are detected most frequently in young smokers [5,6].

The necessity of preventing distal embolic during PCI is becoming increasing recognised, as traditional angioplasty may be associated with distal myocardial necrosis [1].

In our case, angiographic appearance was not suggestive of either obstructive coronary disease or a high embolic risk: none of the criteria set up by Yip et al [7] (cutoff pattern of occlusion, accumulated thrombus > 5 mm proximal to the occlusion, presence of floating thrombus, persistent dye stasis distal to the obstruction, reference lumen diameter of the culprit artery > or = 4 mm, and incomplete obstruction with presence of accumulated thrombus more than three times the reference lumen diameter of culprit artery) was met, and the patient had already received prolonged antiplatelet and anticoagulant therapy.

However, we suspected the presence of a significant thrombotic mass, mainly on the clinical grounds of widespread ECG change and Troponin I elevation, and performed IVUS examination. The importance of the IVUS results is clear: the visualization of a lobulated thrombus, loosely attached to an eroded, non-ruptured plaque, prompted us to use distal protection, which in turn may have prevented significant embolism.

IVUS proved also useful in ensuring full coverage of the lesion, resolving the relation of the lesion with the LAD ostium, correctly sizing the stent, and ruling out thrombus or plaque prolapse through stent struts.

## Conclusion

We present a case of successful management of an intracoronary thrombus, which was accomplished combining IVUS data and a distal protection device.

IVUS proved invaluable in the diagnosis and treatment of this challenging case. We believe IVUS guidance should be considered for assessing thrombotic burden and embolism potential particularly in young patients with ACS when angiographic data are inconclusive.

## Competing interests

The authors declare that they have no competing interests.

## Authors' contributions

IP and AM have written the first draft of the manuscript. AB and IP have performed the coronary intervention. IP, AM, VA and AB participated in the design and coordination of the final manuscript. All authors have read and approved the final manuscript.
